# Simultaneous CD8^+^ T-Cell Immune Response against SARS-Cov-2 S, M, and N Induced by Endogenously Engineered Extracellular Vesicles in Both Spleen and Lungs

**DOI:** 10.3390/vaccines9030240

**Published:** 2021-03-10

**Authors:** Flavia Ferrantelli, Chiara Chiozzini, Francesco Manfredi, Andrea Giovannelli, Patrizia Leone, Maurizio Federico

**Affiliations:** 1 National Center for Global Health, Istituto Superiore di Sanità, Viale Regina Elena 299, 00161 Rome, Italy; flavia.ferrantelli@iss.it (F.F.); chiara.chiozzini@iss.it (C.C.); francesco.manfredi@iss.it (F.M.); patrizia.leone@iss.it (P.L.); 2 National Center for Animal Experimentation and Welfare, Istituto Superiore di Sanità, Viale Regina Elena 299, 00161 Rome, Italy; andrea.giovannelli@iss.it

**Keywords:** SARS-CoV-2, extracellular vesicles, CD8^+^ T cell immunity, Nef

## Abstract

Most advanced vaccines against severe acute respiratory syndrome coronavirus (SARS-CoV)-2 are designed to induce antibodies against spike (S) protein. Differently, we developed an original strategy to induce CD8^+^ T cytotoxic lymphocyte (CTL) immunity based on in vivo engineering of extracellular vesicles (EVs). This is a new vaccination approach based on intramuscular injection of DNA expression vectors coding for a biologically inactive HIV-1 Nef protein (Nef^mut^) with an unusually high efficiency of incorporation into EVs, even when foreign polypeptides are fused to its C-terminus. Nanovesicles containing Nef^mut^-fused antigens released by muscle cells can freely circulate into the body and are internalized by antigen-presenting cells. Therefore, EV-associated antigens can be cross-presented to prime antigen-specific CD8^+^ T-cells. To apply this technology to a strategy of anti-SARS-CoV-2 vaccine, we designed DNA vectors expressing the products of fusion between Nef^mut^ and different viral antigens, namely N- and C-terminal moieties of S (referred to as S1 and S2), M, and N. We provided evidence that all fusion products are efficiently uploaded in EVs. When the respective DNA vectors were injected in mice, a strong antigen-specific CD8^+^ T cell immunity became detectable in spleens and, most important, in lung airways. Co-injection of DNA vectors expressing the diverse SARS-CoV-2 antigens resulted in additive immune responses in both spleen and lungs. Hence, DNA vectors expressing Nef^mut^-based fusion proteins can be proposed for new anti-SARS-CoV-2 vaccine strategies.

## 1. Introduction

Severe acute respiratory syndrome coronavirus (SARS-CoV)-2 first emerged in late 2019 in China [1,2,3]. Globally, the virus has since infected over 72 million individuals and caused more than 1.6 million deaths [4]. Given the severity of the disease, vaccines, and therapeutics to tackle this novel virus are urgently needed.

SARS-CoV-2-related pathology essentially develops in two phases. In the first one, infecting virus replicates in epithelial cells of the upper respiratory tract, generating the first symptoms in approximately 4–7 days. Both B-and T-cell-dependent immune responses are activated within the first week post-symptom onset. Then, the virus can spread towards the lower respiratory tract and lungs, thereby switching on a potent inflammatory response which, for still unknown reasons, may resolve in a fatal disease despite the ongoing immune responses [5].

The majority of SARS-CoV-2 vaccines currently in development aim to induce neutralizing antibodies against spike (S) protein. However, follow-up studies from patients who recovered from a previous epidemic of SARS-CoV suggest that specific antibody responses are short-lived with generation of barely detectable levels of memory B-cells [6,7,8,9,10,11,12,13], and target the primary homologous strain only. Conversely, memory T cells against SARS-CoV persist 11 years post-infection, and have the potential to induce cross-reactive immunity [14,15,16,17,18]. An increasing number of studies shows that also SARS-CoV-2 convalescent patients develop robust T-cell responses [19,20,21,22,23]. Besides S protein, SARS-CoV-2 membrane [M] protein also elicits strong CD8^+^ T cell responses, and a significant reactivity was also reported for both nucleocapsid (N) [19] and ORF1ab [20] proteins. Taken together, this evidence suggests that an efficacious vaccine formulation against SARS-CoV-2 could and should induce strong memory T cell responses against multiple viral antigens. 

Extracellular vesicles (EVs) are a heterogeneous population of membrane nanovesicles, among which exosomes are the most studied [24]. EVs are released by most cell types including myocytes [25], and have key roles in cell-to-cell communication and regulation of immune responses [26,27,28,29]. 

We previously described the ability of Human Immunodeficiency Virus (HIV) -1 Nef to incorporate into EVs released by multiple cell types, including CD4^+^ T lymphocytes and dendritic cells [30]. The incorporation into EVs increases by approximately 100-fold in the case of the Nef G3C-V153L-E177G mutant, most likely due to increased stabilization with cell membranes [31,32]. Moreover, and extremely beneficial for a vaccine, V153L-E177G mutations make Nef^mut^ defective for basically all anti-cellular Nef functions including both CD4 and MHC Class I down-regulation, increased HIV-1 infectivity, and p21 *activated* kinases (PAK)-2 activation [32,33]. Furthermore, we observed that the efficiency of Nef^mut^ incorporation into EVs is maintained even when a foreign protein is fused to its C-terminus [31,32,34,35,36,37]. When DNA vectors expressing Nef^mut^-based fusion proteins are intramuscularly (i.m.) injected in mice, high amounts of the fusion protein are packed into EVs while not altering their spontaneous release from muscle tissue. Nef^mut^-fused antigens released inside muscle-derived-EVs are then internalized by antigen-presenting cells (APCs), which, in turn, cross-present EV content to activate antigen-specific CD8^+^ T cells. These in vivo engineered EVs are assumed to freely circulate into the body, thus having the potential to reach distal tissues. We already documented that they act as an effective vaccine by eliciting potent antigen-specific CTL responses [31,32,37]. Both effectiveness and flexibility of this vaccine platform have been demonstrated with an array of viral products of various origins and sizes, including but not limited to: Human Papilloma Virus (HPV)16-E6 and -E7; Ebola Virus VP24, VP40, and NP; Hepatitis C Virus NS3; West Nile Virus NS3; and Crimean-Congo Hemorrhagic Fever NP [31,37,38]. Of note, in our hands very low to undetectable antigen-specific CD8^+^ T immune responses were observed when animals were injected with DNA vectors expressing the antigen open reading frame (ORF) devoid of the Nef^mut^ sequences [31,37]. The immune response elicited through the Nef^mut^-based platform essentially involves the CTL function in the absence of detectable antibody response [32,37,38].

Here, we tested three SARS-CoV-2 structural antigens, namely spike (S), membrane (M), and nucleocapsid (N) proteins in the context of the Nef^mut^ system. The immunogenicity of DNA vectors expressing each SARS-CoV-2 protein fused with Nef^mut^ and injected in mice either alone or in combination was evaluated in both spleens and lung airways. 

## 2. Materials and Methods

### 2.1. DNA Vector Synthesis

ORFs coding for Nef^mut^ fused with S1, S2, M, or N SARS-CoV-2 proteins were cloned into pVAX1 plasmid (Thermo Fisher, Waltham, MA, USA), i.e., a vector approved by FDA for use in humans. To obtain the pVAX1 vector expressing Nef^mut^, its ORF was cloned into Nhe I and EcoR I sites. To recover vectors expressing Nef^mut^-based fusion products, an intermediate vector referred to as pVAX1/Nef^mut^fusion was produced. Here, the whole Nef^mut^ ORF deprived of its stop codon was followed by a sequence coding a GPGP linker including a unique Apa I restriction site. In this way, sequences comprising the Apa I site at their 5′ end, and the Pme I one at their 3′ end were fused in frame with Nef^mut^ ORF. Stop codons of SARS-CoV-2-related sequences were preceded by sequences coding for a DYKDDDK epitope tag (flag-tag). SARS-CoV-2 sequences were optimized for expression in human cells through GeneSmart software from Genescript. All these vectors were synthesized by Explora Biotech. The pTargeT vector expressing the Nef^mut^/HPV16-E7 fusion protein was already described [38].

### 2.2. Cell Cultures and Transfection

Human embryonic kidney (HEK) 293T cells (ATCC, CRL-11268) were grown in DMEM (Gibco, Thermo Fisher, Waltham, MA) plus 10% heat-inactivated fetal calf serum (FCS, Gibco, Thermo Fisher, Waltham, MA). Transfection assays were performed using Lipofectamine 2000 (Invitrogen, Thermo Fisher Scientific, Waltham, MA)-based method.

### 2.3. EV Isolation

Cells transfected with vectors expressing the Nef^mut^-based fusion proteins were washed 24 h later, and reseeded in medium supplemented with EV-deprived FCS. Supernatants were harvested from 48 to 72 h after transfection. EVs were recovered through differential centrifugations [39] by centrifuging supernatants at 500× *g* for 10 min, and then at 10,000× *g* for 30 min. Supernatants were harvested, filtered with 0.22 μm pore size filters, and ultracentrifuged at 70,000× *g* for l h. Pelleted vesicles were resuspended in 1 × PBS, and ultracentrifuged again at 70,000× *g* for 1 h. Afterwards, pellets containing EVs were resuspended in 1:100 of the initial volume.

### 2.4. Western Blot Analysis

Western blot analyses of both cell lysates and EVs were carried out after resolving samples in 10% sodium dodecyl sulfate-polyacrylamide gel electrophoresis (SDS-PAGE). In brief, the analysis on cell lysates was performed by washing cells twice with 1 × PBS (pH 7.4) and lysing them with 1 × SDS-PAGE sample buffer. Samples were resolved by SDS-PAGE and transferred by electroblotting on a 0.45 μM pore size nitrocellulose membrane (Amersham) overnight (ON) using a Bio-Rad Trans-Blot. EVs were lysed and analyzed as described for cell lysates. For immunoassays, membranes were blocked with 5% non-fat dry milk in PBS containing 0.1% Triton X-100 for 1 h at room temperature, then incubated ON at 4 °C with specific antibodies diluted in PBS containing 0.1% Triton X-100. Filters were revealed using 1:1000-diluted sheep anti-Nef antiserum ARP 444 (MHRC, London, UK), 1:500-diluted anti-β-actin AC-74 mAb from Sigma, 1:500 diluted anti-Alix H-270 polyclonal Abs from Santa Cruz, and 1:1000 diluted anti-flag M2 mAb from Sigma. Filters were analyzed by a Chemi-Doc apparatus, Bio-Rad, and relevant signals were quantified by Image Lab software version 6.1 (Bio-Rad, Hercules, CA, USA).

### 2.5. Mice Immunization

Both 6-weeks old C57 Bl/6 and, for Nef^mut^/S2 immunizations (in view of the lack of already characterized H2^b^ immunodominant S2 epitopes), Balb/c female mice were obtained from Charles River. They were hosted at the Central Animal Facility of the Istituto Superiore di Sanità (ISS), as approved by the Italian Ministry of Health, authorization n. 565/2020 released on 3 June 2020. The day before the first inoculation, microchips from DATAMARS were inserted sub cute at the back of neck between the shoulder blades on the dorsal midline. Preparations of 10 μg for each DNA vector were diluted in 30 μL of sterile 0.9% saline solution. Both quality and quantity of the DNA preparations were checked by 260/280 nm absorbance and electrophoresis assays. Immunization procedures were identical in case of injection with single or multiple vectors. In detail, mice were anesthetized with isoflurane as prescribed in the Ministry authorization. Each inoculum volume was therefore measured by micropipette, loaded singly into a 1 mL syringe without dead volume, and injected into mouse quadriceps. Immediately after inoculation, mice underwent electroporation at the site of injection through the Agilpulse BTX device using a 4-needle array 4 mm gap, 5 mm needle length, with the following parameters: 1 pulse of 450 V for 50 µs; 0.2 ms interval; 1 pulse of 450 V for 50 µs; 50 ms interval; 8 pulses of 110 V for 10 ms with 20 ms intervals. The same procedure was repeated for both quadriceps of each mouse. Immunizations were repeated after 14 days. Fourteen days after the second immunization, mice were sacrificed by either cervical dislocation or CO_2_ inhalation, following the recommendations included in the Ministry authorization protocol.

### 2.6. Cell Isolation from Immunized Mice

Spleens were explanted by qualified personnel of ISS Central Animal Facility, and placed into a 2 mL Eppendorf tubes filled with 1 mL of RPMI 1640 (Gibco, Thermo Fisher, Waltham, MA, USA), 50 µM 2-mercaptoethanol (Sigma, St. Louis, MI, USA). Spleens were transferred into a 60 mm Petri dish containing 2 mL of RPMI 1640 (Gibco, Thermo Fisher, Waltham, MA, USA), 50 µM 2-mercaptoethanol (Sigma, St. Louis, MI, USA). Splenocytes were extracted by incising the spleen with sterile scissors and pressing the cells out of the spleen sac with the plunger seal of a 1 mL syringe. After addition of 2 mL of RPMI medium, cells were transferred into a 15 mL conical tube, and the Petri plate was washed with 4 mL of medium to collect the remaining cells. After a three-minute sedimentation, splenocytes were transferred to a new sterile tube to remove cell/tissue debris. Counts of live cells were carried out by the trypan blue exclusion method. Fresh splenocytes were resuspended in RPMI complete medium, containing 50 µM 2-mercaptoethanol and 10% FBS, and tested by IFN-γ EliSpot assay. 

For bronchoalveolar lavages, mice were sacrificed by CO_2_ inhalation, placed on their back, and dampened with 70% ethanol. Neck skin was opened to the muscles by scissors, and muscles around the neck and salivary glands were gently pulled aside with forceps to expose the trachea. A 15 cm long surgical thread was then placed around the trachea and a small hole was cut by fine point scissors between two cartilage rings. A 22 G × 1” Exel Safelet catheter was carefully inserted about 0.5 cm into the hole, and then the catheter and the trachea were firmly tied together with the suture. A 1 mL syringe was loaded with 1 mL of cold PBS and connected to the catheter. The buffered solution was gently injected into the lungs and aspirated while massaging mouse thorax. Lavage fluid was transferred to a 15 mL conical tube on ice, and lavage repeated two more times [40,41] Total lavage volume was approximately 2.5 mL/mouse. Cells were recovered by centrifugation, resuspeded in cell culture medium, and counted.

### 2.7. IFN-γ EliSpot Analysis

A total of 2.5 × 10^5^ live cells were seeded in each IFN-γ EliSpot microwell. Cultures were run in triplicate in EliSpot multiwell plates (Millipore, cat n. MSPS4510) pre-coated with the AN18 mAb against mouse IFN-γ (Mabtech, Nacka Strand, Sweden) in RPMI 1640 (Gibco, Thermo Fisher, Waltham, MA, USA), 10% FCS, 50 µM 2-mercaptoethanol (Sigma, St. Louis, MI, USA) for 16 h in the presence of 5 µg/mL of the following CD8-specific peptides: HPV-16 E7 (H2-K^b^): 21–28 DLYCYEQL [42]; 49–57 RAHYNIVTF [42]; 67–75 LCVQSTHVD [43]. SARS-CoV-2 S1 (H2-K^b^): 525–531 VNFNFNGL [44]; SARS-CoV-2 S2 (H2-K^d^): 1079–1089 PAICHDGKAH [45]; SARS-CoV-2 M (H2-K^b^): 173–180 RTLSYYKL [46]; SARS-CoV-2 N (H2-K^b^): 219–228 ALALLLLDRL [46]. As negative controls, 5 µg/mL of either H2-K^b^ or H2-K^d^-binding peptides were used. More than 70% pure preparations of the peptides were obtained from Elabioscences. SARS-CoV-2 peptide collections were obtained from BEI resources. Peptide pools were adjusted at the concentration of 50 μg/mL for each peptide, and used at final concentration of 1 μg/mL each. For cell activation control, cultures were treated with 10 ng/mL phorbol 12-myristate 13-acetate PMA (Sigma, St. Louis, MI, USA) plus 500 ng/mL of ionomycin (Sigma, St. Louis, MI, USA). PeptTivator NS3 (Miltenyi, Bergish Gladbach, Germany) (i.e., a pool of HCV genotype 1b NS3 peptides consisting of 15-mers with 11 aa overlap covering the whole NS3 sequence) was used as negative control. After 16 h, cultures were removed, and wells incubated with 100 µL of 1 µg/mL of the R4-6A2 biotinylated anti-IFN-γ (Mabtech, Nacka Strand, Sweden) for 2 h at r.t. Wells were then washed and treated for 1 h at r.t. with 1:1000 diluted streptavidine-ALP preparations from Mabtech. After washing, spots were developed by adding 100 µL/well of SigmaFast BCIP/NBT, Cat. N. B5655. Spot-forming cells were finally analyzed and counted using an AELVIS EliSpot reader.

### 2.8. Cell Staining 

Cells from bronchoalveaolar lavage fluids (BALFs) were seeded (2 × 10^6^/mL) in RPMI medium, 10% FCS, 50 µM 2-mercaptoethanol (Sigma, St. Louis, MI, USA), and 1 μg/mL brefeldin A (BD Biosciences). Control conditions were carried out either by adding 10 ng/mL PMA (Sigma, St. Louis, MI, USA) and 1 μg/mL ionomycin (Sigma, St. Louis, MI, USA), or with unrelated peptides. After 16 h, cultures were stained with 1 μL of LIVE/DEAD Fixable Aqua Dead Cell reagent (Invitrogen ThermoFisher, Waltham, MA) in 1 mL of PBS for 30 miN at 4 °C and washed twice with 500 μL of PBS. To minimize non-specific staining, cells were pre-incubated with 0.5 μg of Fc blocking mAbs (i.e., anti-CD16/CD32 antibodies, Invitrogen/eBioscience Thermo Fisher, Waltham, MA) in 100 μL of PBS with 2% FCS for 15 min at 4 °C. For the detection of cell surface markers, cells were stained with 2 μL of the following Abs: FITC-conjugated anti-mouse CD3, APC-Cy7-conjugated anti-mouse CD8a, and PerCP-conjugated anti-mouse CD4 (BD Biosciences, Franklin Lakes, NJ, USA) and incubated for 1 h at 4 °C. After washing, cells were permeabilized and fixed through the Cytofix/Cytoperm kit (BD Biosciences, Franklin Lakes, NJ, USA) as per the manufacturer’s recommendations and stained for 1 h at 4 °C with 2 μL of the following Abs: PE-Cy7-conjugated anti-mouse IFN-γ, PE-conjugated anti-mouse IL-2 (Invitrogen/eBioscience Thermo Fisher, Waltham, MA/eBioscience), and BV421 rat anti-mouse TNF-α BD Biosciences in a total of 100 μL of 1× Perm/Wash Buffer (BD Biosciences, Franklin Lakes NJ, USA). After two washes, cells were fixed in 200 μL of 1× PBS/formaldehyde (2% v/v). Samples were then assessed by a Gallios flow cytometer and analyzed using Kaluza software (Beckman Coulter, Brea, CA, USA). Gating strategy was as follows: live cells as detected by Aqua LIVE/DEAD Dye vs. FSC-A, singlet cells from FSC-A vs. FSC-H (singlet 1) and SSC-A vs. SSC-W (singlet 2), CD3-positive cells from CD3- (FITC) vs. SSC-A-, CD8-, or CD4-positive cells from CD8 (APC-Cy7) vs. CD4 (PerCP). The CD8^+^ cell population was gated against APC-Cy7, PE, and BV421 to observe changes in IFN-γ, IL-2, and TNF-α production, respectively. Boolean gates were created in order to determine any cytokine co-expression pattern.

### 2.9. Statistical Analysis

When appropriate, data are presented as mean + standard deviation (SD). In some instances, the Mann–Whitney U test was used. *p* < 0.05 was considered significant.

## 3. Results

### 3.1. Construction of Vectors Expressing Nef^mut^ Fused with SARS-Cov-2 Antigens

**Figure 1:**
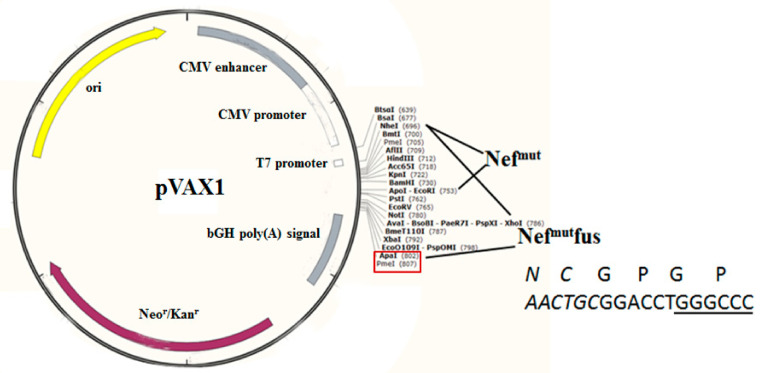
Map of vectors expressing SARS-CoV-2-based fusion proteins. Shown are the map of pVAX1 vector as well as the cloning strategies pursued to obtain both pVAX1-Nef^mut^ and pVAX1-Nef^mut^fusion vectors. Restriction sites where the SARS-CoV-2 ORFs were inserted are highlighted in the red box. On the bottom right, shown are both amino acid and nucleotide sequences of Nef^mut^ C-terminus in the pVAX1-Nef^mut^fusion vector (in italics), as well as of the GPGP linker overlapping the Apa I restriction site (underlined sequence).

All Nef^mut^/SARS-CoV-2 fusion proteins were expressed in the context of the pVAX1 vector (Figure 1). 

**Figure 2:**
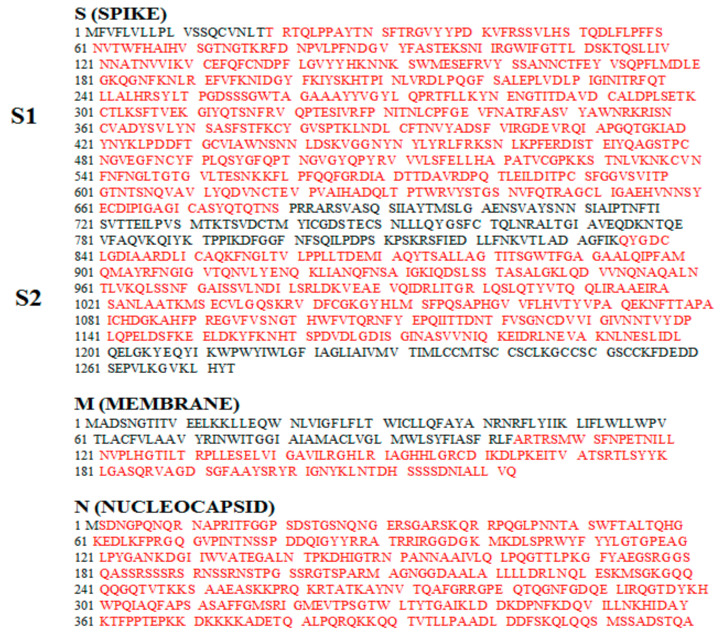
Amino acid sequences of S, N, and M proteins from the Italian clinical isolate of SARS-CoV-2 ITA/INMI1/2020 (https://www.ncbi.nlm.nih.gov/nuccore/MT066156; GenBank: MT066156.1, https://www.ncbi.nlm.nih.gov/nuccore/MT066156;%20GenBank:%20MT066156.1). Sequences included in the Nef^mut^-based vectors are highlighted in red.

ORFs encoding SARS-CoV-2 S, M, and N proteins were from the Italian clinical isolate ITA/INMI1/2020. Each Nef^mut^-fusion construct has been designed to guarantee optimal internalization efficiency into EVs considering both size of foreign sequences and presence of hydrophobic domains, which could interfere with the intraluminal localization. Furthermore, already characterized mouse CD8^+^ T cell immunodominant epitopes were preserved [44,45,46]. SARS-CoV-2-related amino acid sequences included in the Nef^mut^-based fusion proteins are highlighted in Figure 2. 

Soon after cell attachment, SARS-CoV-2 S is cleaved at the furin-like site PRRARS, which identifies the boundary between S1 and S2 subunits [47]. We predicted that possible intracellular, furin-dependent cleavage of S would negatively affect the uploading of the entire S protein fused with Nef^mut^ into EVs. To overcome this limitation, we designed two Nef^mut^-based constructs, i.e., Nef^mut^-S1 (aa 19 to 680), where both signal peptide and furin-like cleavage site were excluded, and Nef^mut^-S2 (aa 836 to 1196), including the extracellular portion of the protein with the exclusion of the two fusion domains, the transmembrane region, and the short intracytoplasmic tail [48,49]. 

SARS-CoV-2 M protein (221 aa) is composed of an amino-terminal exterior region of 18 amino acids, a transmembrane region accounting for approximately one third of the entire protein, and a C-terminal region composed of 123 intraluminal residues [47]. To guarantee an efficient uploading into EV lumen, only the C-terminal region of M (aa 94 to 221) was fused to Nef^mut^. 

Finally, the full length ORF of the N protein (422 aa), except for M1 amino acid, was fused to Nef^mut^. 

### 3.2. Uploading of Nef^mut^-Based Products of Fusion with SARS-Cov-2 Antigens in Evs

Cell expression of the products of fusion between Nef^mut^ and SARS-CoV-2 antigens S1, S2, M, and N was evaluated by transient transfection in HEK293T cells. To inspect the uploading into EVs of the fusion products, supernatants from transfected cells were collected 48-72 h after transfection, and then processed by differential centrifugations. 

**Figure 3:**
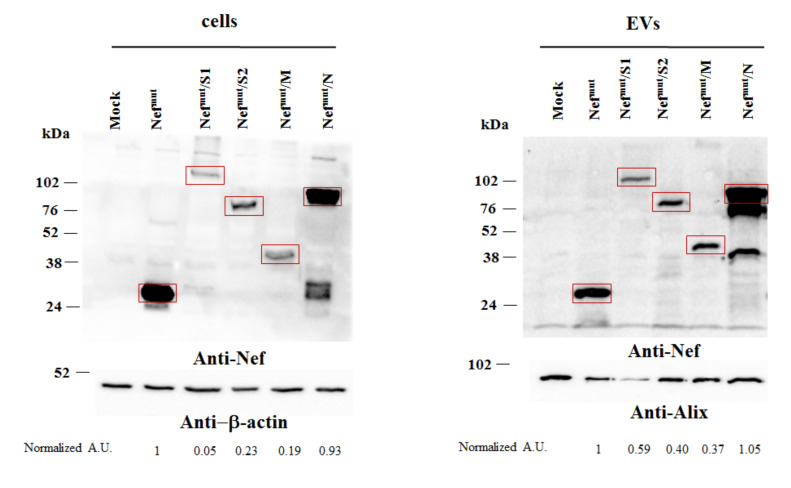
Detection of Nef^mut^/SARS-CoV-2 fusion products in transfected cells and extracellular vesicles (EVs). Western blot analysis of 30 μg of lysates from HEK293T cells transfected with DNA vectors expressing Nef^mut^ fused with either S1, S2, M, or N SARS-CoV-2 open reading frames (ORFs) (left panels). Equal volumes of buffer where purified EVs were resuspended after differential centrifugations of respective supernatants were also analyzed (right panels). As control, conditions from mock-transfected cells as well as cells transfected with a vector expressing Nef^mut^ alone were included. Polyclonal anti-Nef Abs served to detect Nef^mut^-based products, while β-actin and Alix were revealed as markers for cell lysates and EVs, respectively. Relevant signals are highlighted. Molecular markers are given in kDa. Relative intensities of relevant signals after normalization with either β-actin (for cell lysates) or Alix (for EVs) signals were also reported as arbitrary units (A.U.), and considering the Nef^mut^ conditions as reference samples. The results are representative of four independent experiments (Figure S1).

Both cell lysates and EVs isolated from respective supernatants were analyzed by western blot assay (Figure 3 and Supplementary Figure S1). Upon incubation with anti-Nef Abs, the cell-associated steady-state levels of all Nef^mut^-derivatives were clearly detectable. The strongest signals appeared in lysates of cells expressing Nef^mut^ alone and those expressing its product of fusion with N. In the latter case, products of lower molecular weight were also detectable, possibly originated by intracellular cleavage. 

The results we obtained from the analysis of EVs basically reflected those from cell lysate analysis (Figure 3 and Figure S1). Also, in this case, the presence of apparently full-length Nef^mut^/N fusion product coupled with that of two products of lower molecular weight.

Taken together, these results indicated that all fusion products we designed are stable and associate with EVs.

### 3.3. Detection of Virus-Specific CD8^+^ T Cells in Spleens from Mice Injected with Vectors Expressing Nef^mut^ Fused with Each SARS-Cov-2 Antigen

**Figure 4:**
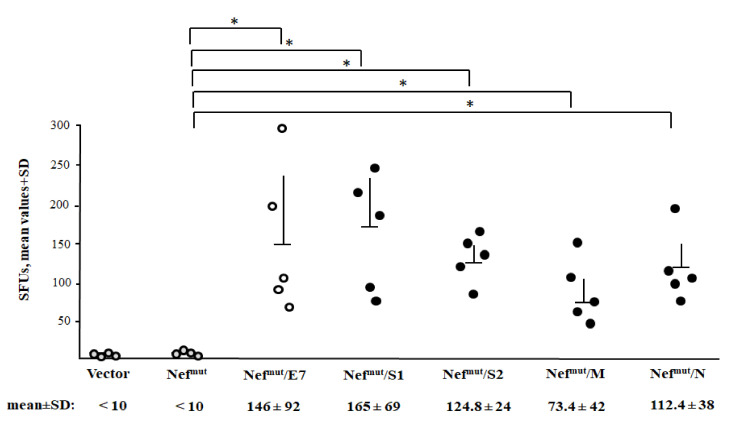
SARS-CoV-2-specific CD8^+^ T cell immunity as detected in spleens from mice i.m. injected with Nef^mut^-derived DNA vectors. CD8^+^ T cell immune response in either C57 Bl/6 or, for immunization with Nef^mut^-S2-expressing vector only, Balb/c mice inoculated i.m. with DNA vectors expressing Nef^mut^ either alone (4 mice) or fused with the indicated SARS-CoV-2 antigens (5 mice per group). As controls, mice were inoculated with either void vector (four mice) or, for C57 Bl/6 mice only, a vector expressing Nef^mut^/E7 (five mice). At the time of sacrifice, 2.5 × 10^5^ splenocytes were incubated ON with or without 5 μg/ml of either unrelated or SARS-CoV-2-specific peptides in triplicate IFN-γ EliSpot microwells. Shown are the numbers of IFN-γ spot-forming units (SFU)/well calculated as mean values of triplicates after subtraction of mean spot numbers calculated in wells of splenocytes treated with unspecific peptides. Reported are intragroup mean values + standard deviations also. The SARS-CoV-2-specific immune response in Balb/c mice injected with either void or Nef^mut^ expressing vectors remained at background levels, i.e., the levels measured in wells treated with unspecific peptides (not shown). Circles sign values measured in cultures of splenocytes isolated from each injected mouse. The results are representative of two independent experiments. Statistical significance compared to values obtained with splenocytes injected with Nef^mut^ expressing vector was determined by Mann–Whitney U test. * *p* < 0.05.

Next, we evaluated the immunogenicity of each SARS-CoV-2 antigen fused with Nef^mut^ as expressed by the different DNA vectors. As benchmark, mice were immunized with a vector expressing Nef^mut^/E7, i.e., a vector whose injection generated a both strong and effective anti-E7 CTL immune response [37,50]. Either C57 Bl/6 or, in the case of immunization with the Nef^mut^/S2 vector, Balb/c mice were i.m. inoculated in each quadriceps with 10 μg of each DNA vector and, as control, equal amounts of either void or pVAX1-Nef^mut^ vector. Injections were immediately followed by electroporation procedures. Fourteen days after the second immunizations, splenocytes were tested by IFN-γ EliSpot assay. Octo-decamers specific for CD8^+^ T cell immunodominant epitopes previously described for SARS-CoV, and whose sequences are conserved in SARS-CoV-2, were used. Sustained antigen-specific CD8^+^ T cell immune responses were observed in mice injected with every DNA vector expressing Nef^mut^-based fusion proteins (Figure 4). Although the assay does not allow a rigorous quantification of the immune response, the CD8^+^ T cell activation extents detected in mice injected with vectors expressing SARS-CoV-2-derivatives appeared comparable to that induced by the Nef^mut^/E7-expressing vector we considered as a “gold-standard”.

These data indicated that the DNA vectors expressing the four SARS-CoV-2-based fusion proteins were able to elicit a virus-specific CD8^+^ T cell immunity.

### 3.4. Detection of SARS-Cov-2-Specific CD8^+^ T Lymphocytes in Cells from Bronchoalveolar Lavages of Immunized Mice

The induction of a CD8^+^ T cell immune response at the pulmonary tissues should be considered a mandatory feature for any CD8^+^ T cell-based vaccine against respiratory diseases. In general, the immune response against respiratory viruses leads to formation of three distinct antigen-specific CD8^+^ T cell populations: circulating effector memory (T_EM_); central memory (T_CM_), basically residing in secondary lymphoid organs; and resident memory (T_RM_) cells in peripheral tissues [51]. In lungs, these latter are considered a self-renewing cell population only minimally replenished by circulating T_EM_ [52]. On this basis, the presence of virus-specific CD8^+^ T cells in spleens would not necessarily guarantee adequate levels of immunity at lung tissues, i.e., the district directly involved in SARS-CoV-2 pathogenesis.

**Figure 5:**
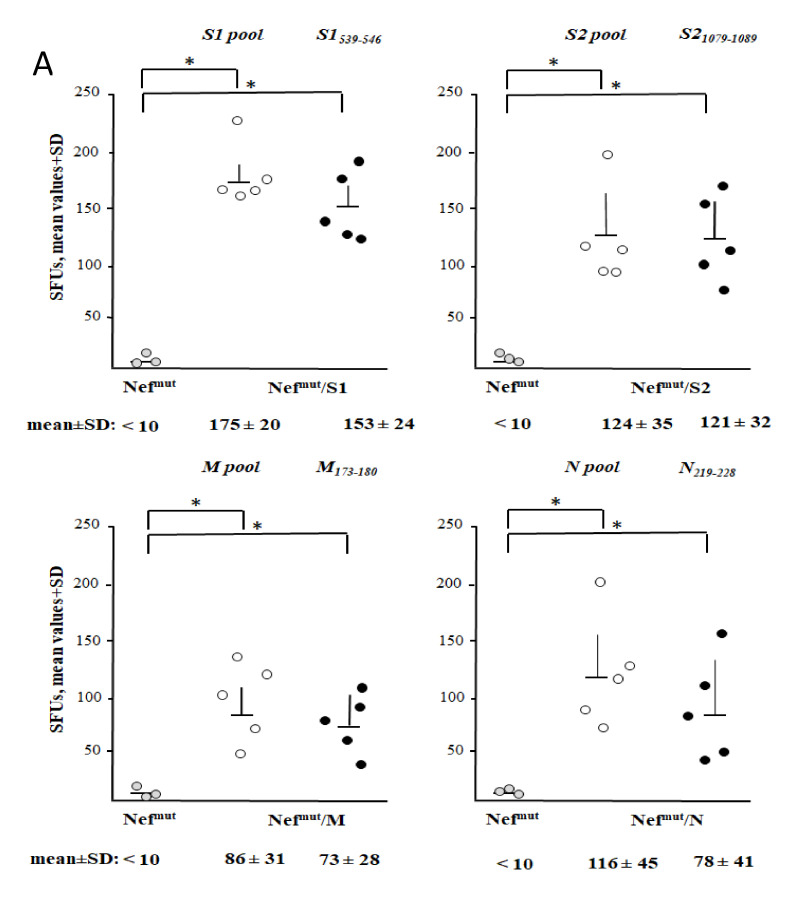

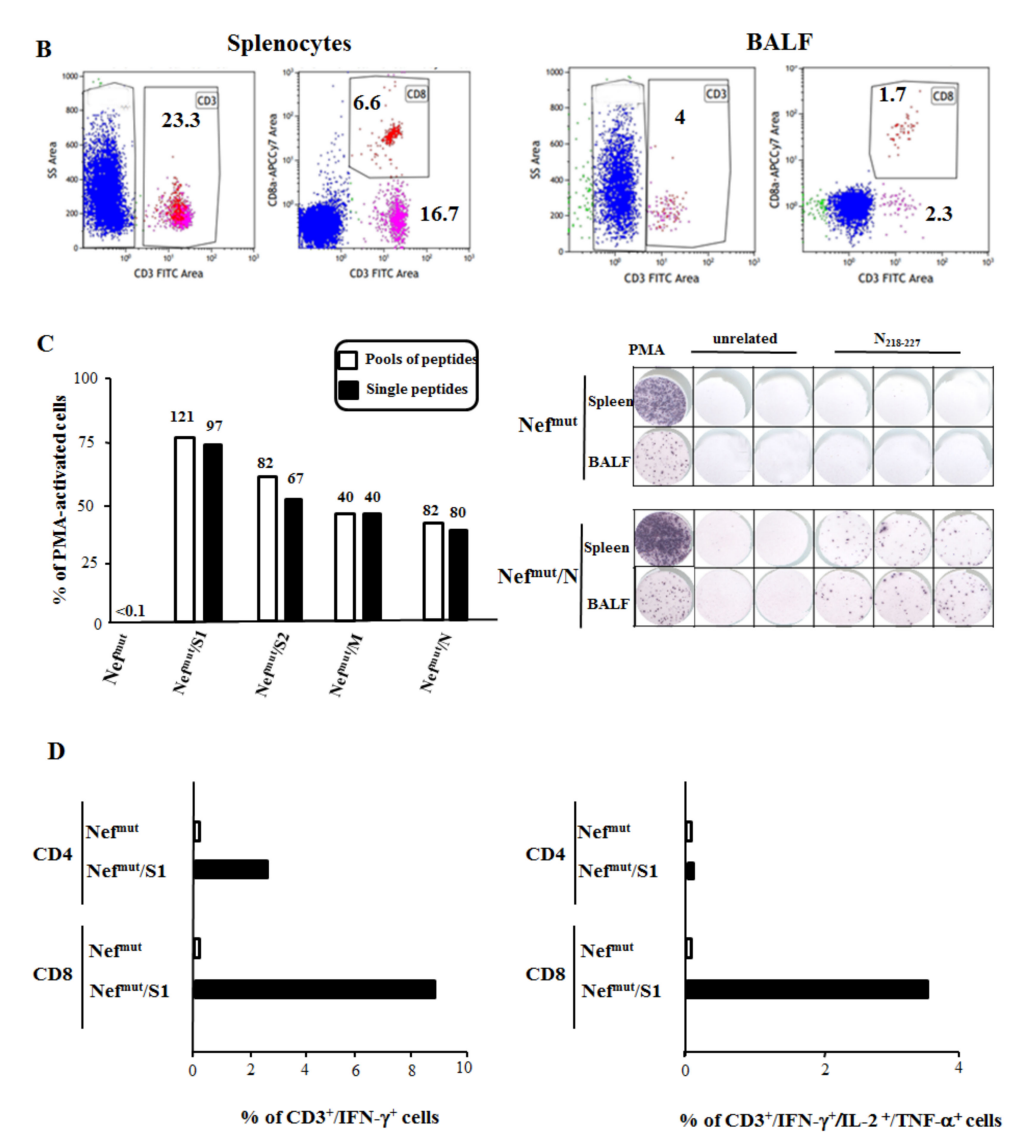
SARS-CoV-2 -specific total cell and CD8^+^ T cell immunity in both spleens and bronchoalveaolar lavage fluids (BALFs) of mice immunized i.m. by Nef^mut^-derived DNA vectors. (**A**) Total cell and CD8^+^ T cell immune responses in C57 Bl/6 and, for immunization with Nef^mut^/S2 expressing vector only, Balb/c mice inoculated i.m. with DNA vectors expressing Nef^mut^ either alone (three mice), or fused with the indicated SARS-CoV-2 antigens (five mice per group). Total cell immune responses were measured using pools of 13- to 17-mers, whereas single octo-decamers were used to evaluate the CD8^+^ T cell immune responses. At the time of sacrifice, 2.5 × 10^5^ splenocytes were incubated ON with either unrelated or SARS-CoV-2-specific peptides in triplicate IFN-γ EliSpot microwells. Shown are the numbers of SFUs/well as mean values of triplicates after subtraction of mean spot numbers measured in wells of splenocytes treated with unspecific peptides. Reported are intragroup mean values + standard deviations also. No virus-specific immune responses were detected using a pool of peptides from a heterologous viral product, i.e., HCV-NS3 (not shown). Circles sign values measured in cultures of splenocytes isolated from each injected mouse. The results are representative of two independent experiments. Statistical significance compared to values obtained with splenocytes from mice injected with Nef^mut^ expressing vector was determined by Mann–Whitney U test. * *p* < 0.05. (**B**) FACS analysis of cell populations recovered through BALFs (right panels) compared to those from spleens (left panels). Total lymphocytes were identified through anti-CD3 labeling among which both CD4^+^ and CD8^+^ sub-populations were distinguished. Percentages of CD3^+^, CD4^+^, and CD8^+^ cells over the total of analyzed events are indicated. The data refer to mice injected with the Nef^mut^/N expressing vector, and are representative of eight independent analyses. (**C**) Percentages of spot-forming units (SFUs) detected in IFN-γ EliSpot microwells seeded with 10^5^ cells isolated from pooled BALFs and cultivated in the presence of virus-specific peptides, as calculated relatively to SFUs scored in microwells seeded with an equal number of cells treated with PMA plus ionomycin. Cell activation was assayed using either peptides pools or single peptides. Cell samples seeded with unrelated peptides scored at background levels (not shown). Absolute SFU numbers are also indicated. The results are representative of two independent experiments. On the right, shown are raw data from a representative IFN-γ EliSpot plate where cells from both spleens and BALFs of mice injected with DNA vectors expressing either Nef^mut^ or Nef^mut^/N were stimulated with either PMA plus ionomycin, an unrelated peptide, or the N specific peptide. (**D**) Intracellular accumulation of IFN-γ and IFN-γ, IL-2, and TNF-α in both CD8^+^ T and CD4^+^ T cells from BALFs of mice injected with vectors expressing either Nef^mut^ or Nef^mut^/S1. Cells isolated from BALFs of five injected mice were pooled, and incubated ON with either the pool of S1 peptides, or a peptide pool of HCV-NS3 at final concentration of 1 μg/mL for each peptide and in the presence of brefeldin A. Therefore, cells were analyzed by ICS. Shown are percentages of cytokine-expressing CD3^+^/CD4^+^ and CD3^+^/CD8^+^ T cells after subtraction of values measured in cultures treated with the pool of HCV-NS3 peptides. The results are representative of two independent experiments.

The here above immunogenicity experiment was reproduced with the specific aim to test the cell immune response at the level of lung airways. To this end, mice were immunized as described and, 14 days after the second immunization, cells from both spleens and BALFs were isolated and tested in IFN-γ EliSpot assay. Both SARS-CoV-2-related pools of peptides to test the total cell immune response, and octo-decamers specific for the CD8^+^ T cell immune response, were used. Results obtained with splenocytes (Figure 5A) fairly reproduced those described in the previous immunogenicity experiment. The higher responses detected with peptide pools were likely a consequence of a co-induced CD4^+^ T cell immune response. 

The immune responses in cells isolated from BALFs (Figure 5B) were evaluated in terms of percentages of SFUs measured upon peptide stimulation over those counted in PMA-stimulated cultures. Overall, we found quite high percentages of SARS-CoV-2 specific CD8^+^ T cells compared to the number of PMA-activated cells (Figure 5C). The strongest immune responses appeared in S1-immunized mice. Using peptide pools, the immune responses appeared only incrementally increased compared to what was observed with single peptides, likely consequence of a predominant CD8^+^ T cell immunity. This hypothesis was supported by data obtained through intracellular staining (ICS) and FACS analysis of cells from BALFs of mice immunized by the Nef^mut^/S1 expressing vector (Figure 5D). In fact, a prevalent activation of CD8^+^ T over CD4^+^ T cells was observed after stimulation with the pool of S1 peptides. Of note, a significant subpopulation of activated CD8^+^ T cells co-expressed IFN-γ, IL-2, and TNF-α, indicating the induction of polyfunctional CD8^+^ T cells (Figure 5D).

In conclusion, immunization with Nef^mut^-based DNA vectors generated a strong antiviral CD8^+^ T cell immune response also in lung airways, i.e., the peripheral tissue most critically involved in the virus-induced respiratory disease.

### 3.5. Additive Immunogenic Effect in Mice Co-Injected with DNA Vectors Expressing Diverse SARS-Cov-2–Based Fusion Proteins

**Figure 6:**
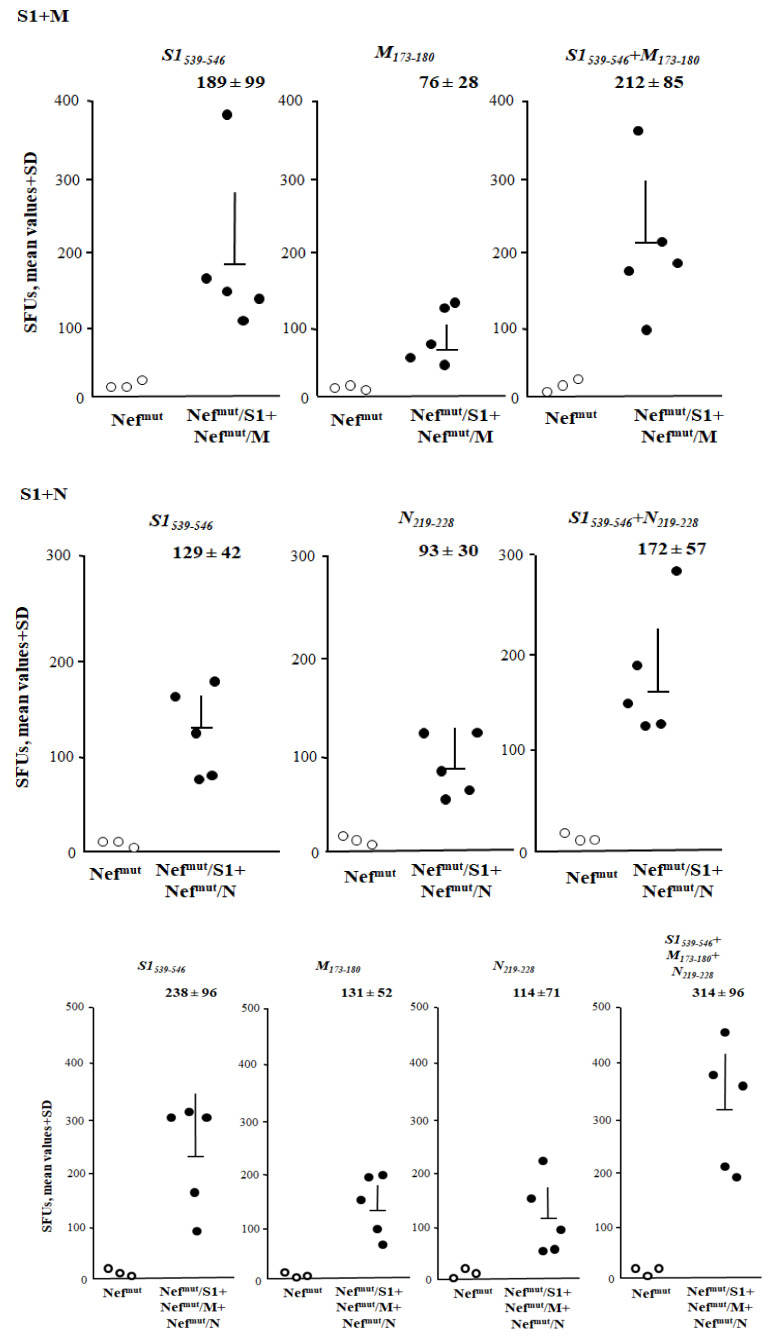
SARS-CoV-2-specific CD8^+^ T cell immune responses induced in spleens from mice immunized by combination of Nef^mut^-based DNA vectors. CD8^+^ T cell immune response in C57 Bl/6 mice inoculated i.m. with either the DNA vector expressing Nef^mut^ alone (3 mice) or the indicated combinations of vectors expressing Nef^mut^ fused with the SARS-CoV-2 antigens (5 mice per group). The CD8^+^ T cell immune responses were measured using the indicated peptides and combination thereof. At the time of sacrifice, 2.5 × 10^5^ splenocytes were incubated overnight (ON) with or without 5 μg/mL of either unrelated or SARS-CoV-2-specific peptides in triplicate IFN-γ EliSpot microwells. Shown are the numbers of SFU/well calculated as mean values of triplicates after subtraction of mean values measured in wells of splenocytes treated with unspecific peptides. Reported are intragroup mean values + standard deviations also. Circles sign values measured in cultures of splenocytes isolated from each injected mouse The results are representative of two independent experiments.

An intrinsic value added of the Nef^mut^-based CTL vaccine platform consists of the possibility to immunize against different antigens through a single DNA injection. Co-immunizations were attempted by injecting mice with combinations of the Nef^mut^-based DNA vectors expressing SARS-CoV-2 antigens. In particular, mice were immunized with combinations of DNA vectors expressing S1, M and N. Fourteen days after the second immunization, cells from both spleens and BALFs were isolated and tested for the presence of SARS-CoV-2-specific CD8^+^ T cells. Concerning the splenocytes cultures, immune responses of potencies similar to those previously observed in mice injected with single DNA vectors were sensed by the treatment with single specific peptides (Figure 6). When combinations of peptides specific for the different viral antigens were used, the resulting CD8^+^ T cell activation appeared consistently higher than that observed using single peptides (Figure 6). The highest immune response was detected in cultures of splenocytes from triple injected mice tested with peptides specific for each SARS-CoV-2 antigen.

**Figure 7:**
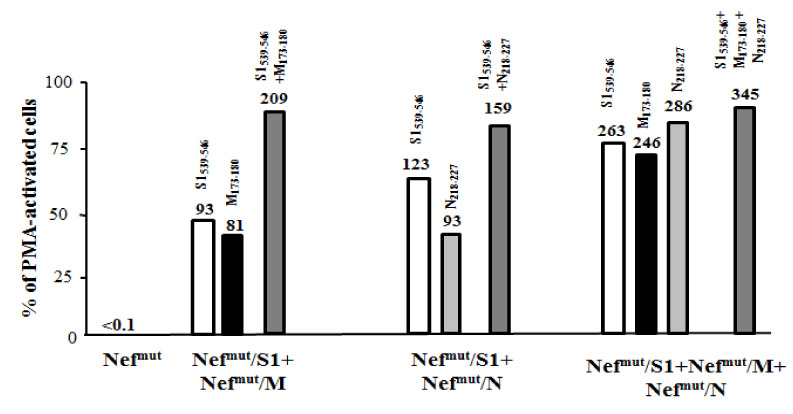
SARS-CoV-2-specific CD8^+^ T cell immune responses in cells from BALFs of mice immunized by combination of Nef^mut^-based DNA vectors. Shown are the percentages of SFUs detected in IFN-γ EliSpot microwells seeded with 10^5^ cells from BALFs pooled from mice injected with the indicated combinations of DNA vectors in the presence of virus-specific peptides over SFUs scored in PMA plus ionomycin treated cells. Conditions comprising either single peptides or combinations thereof were tested. Samples tested in the presence of unrelated peptides scored at background levels. Absolute SFU numbers are also indicated. The results are representative of two independent experiments.

Also, in lung airways, the anti-SARS-CoV-2 CD8^+^ T cell immune response benefited from DNA combinations (Figure 7). Accordingly with what was observed in spleens, the strongest response was detected in cells from mice co-injected with S1-, M-, and N-expressing vectors.

The induction of high levels of virus-specific CD8^+^ T cells in lung airways can be considered a strong benefit for the here proposed anti-SARS-CoV-2 vaccine strategy. More in general, these data open the way towards the development of CD8^+^ T cell vaccination strategies against multiple antigens of the same and, theoretically, different pathogens also. 

## 4. Discussion

Both experimental and clinical evidence already demonstrated the key role of CTLs in the mechanism of protection induced by a number of vaccine preparations against acutely infecting viruses [53,54,55,56,57,58,59,60,61,62,63,64,65,66,67,68]. For instance, in non-human primates, the vaccine-induced immunity against Ebola virus is marked by the protective effect mediated by CD8^+^ T-cells [69]. Similarly, the key role of CD8^+^ T cell-mediated immune response in vaccine-induced protection against West Nile Virus has been demonstrated [70,71]. Also, in the case of infections by human Coronaviruses, the CD8^+^ T-cell immunity plays a relevant role in the recovery from SARS-CoV, MERS-CoV [72], and SARS-CoV-2 [20] infections.

Although the correlates of protection against SARS-CoV-2 are still unknown, the majority of current efforts towards the development of SARS-CoV-2 vaccines are focused on the induction of antibodies against the S protein. However, results from studies on the related SARS-CoV indicated that the virus-specific antibody response was short-lived. In particular, virus-specific IgM and IgA lasted less than six months, while virus-specific IgG titers peaked four months post-infection, and markedly declined after one year at best [6,7,8,9,10,11,12]. The risk of losing this protection over time was further indicated in a six-year follow-up study showing the absence of peripheral memory B cell responses in recovered SARS patients [13].

Another potential obstacle for SARS-CoV-2 vaccine development is the risk of triggering antibody-dependent enhancement (ADE) of virus infection. Vaccine-induced ADE has been documented in the case of SARS-CoV infections [71,72,73], and just recently suggested for SARS-CoV-2 [74]. Thus, without a full understanding of the mechanisms underlying protective immunity, many fear that some vaccines might worsen the disease rather than prevent it, echoing the disastrous effects of the Dengvaxia tetravalent yellow fever-dengue antibody-generating vaccine [75].

Following a prime-boost immunization approach, Channappanavar and coll. showed that CD8^+^ T-cells specific for a single SARS-CoV immunodominant epitope protected mice from an otherwise lethal dose of virus in the absence of neutralizing antibodies [76]. The same study demonstrated the lack of memory CD8^+^ T-cell-mediated immunopathology, suggesting that the induction of these cells was safe. On the other hand, unlike patient waning serum antibody levels, CD8^+^ T-cell responses against S and N proteins can still be detected in peripheral blood of recovered SARS-CoV patients even 11 years post-infection [13,14,15,16,17,18]. Grifoni and coll. demonstrated robust T cell responses against S, M, and N proteins in 20 COVID-19 convalescent patients [19]. Circulating SARS-CoV-2-specific CD8^+^ and CD4^+^ T cells were found in 70% and 100% of patients, respectively [19]. Another study identified the most part of SARS-CoV-2-specific epitopes recognized by memory CD8^+^ T cells from COVID-19 patients in both N and ORF1ab proteins [20]. Taken together, this evidence suggests that a strong memory CD8^+^ T-cell response should be a component of any vaccine regimen for human CoVs, possibly in combination with immunogens inducing safe neutralizing antibodies.

As from previous immunogenicity studies with DNA vectors expressing Nef^mut^ fused with several viral and tumor antigens, HPV-16 E7 reproducibly appeared the antigen eliciting the most potent CD8^+^ T cell response [31,37]. Here, we provide evidence that DNA vectors expressing single SARS-CoV-2 antigens elicited a CD8^+^ T cell immune response at least comparable to that induced by Nef^mut^/E7 [37]. Most striking, valuable levels of virus-specific CD8^+^ T cells were identified in cells from BALFs of immunized mice. Resident CD8^+^ T cells in lungs are basically maintained independently of the pool of circulating CD8^+^ T cells, undergoing homeostatic proliferation to replenish continuous loss of cells through intraepithelial migration towards lung airways. In view of the quite high percentages of virus-specific CD8^+^ T cells we detected in BALFs, it is conceivable that they were generated by activation occurred at local, e.g., mediastinal, lymph nodes rather than diffusion of circulating virus-specific CD8^+^ T cells. This hypothesis is consistent with the biological properties of immunogenic EVs, which are expected to freely circulate into the body, thereby targeting spleen, liver, and lungs, as already documented [77]. Upon EV capture, tissue-resident APCs would migrate to local lymph nodes, thereby switching the processes leading to antigen-specific CD8^+^ T cell activation. Even if the underlying mechanism needs to be elucidated, the results we obtained with BALFs should be considered of relevance for a cell-mediated vaccine strategy conceived to battle a respiratory virus.

The immune response we observed by co-injecting different DNA vectors was reproducibly higher than that obtained by injecting each DNA vector alone. No negative interferences among the diverse antigens appeared by testing the immune response with cells isolated from spleens and, most important, BALFs. Hence, the Nef^mut^-based vaccine platform offers the option to elicit a simultaneous CD8^+^ T cell response against different antigens, which was proven to be effective in peripheral tissues as well. 

The major limitation of our study consists of the lack of efficacy data from experiments of virus challenge. In particular, it would be of interest in evaluating the antiviral effect induced in hACE-transgenic mice by Nef^mut^-derived products compared to that produced by most advanced anti-SARS-CoV-2 vaccines. Also, a conclusive phenotypic and functional characterization of SARS-CoV-2 specific CD8^+^ T cells detected in BALFs from immunized mice would be highly informative regarding their origin and intrinsic antiviral activity. On this subject, however, the proof that a consistent fraction of virus-specific CD8^+^ T cells upon peptide stimulation co-expressed IFN-γ, IL-2 and TNF-α was strongly suggestive of the generation of CD8^+^ T cells with potential CTL antiviral activity. 

Notwithstanding, here presented studies led to a number of original achievements, in particular: (i) the demonstration that DNA vectors expressing either SARS-CoV-2 S1, S2, M or N proteins fused with Nef^mut^ elicit a strong CD8^+^ T immunity; (ii) the evidence that this immune response develops in lung airways also, and (iii) the proof that the CD8^+^ T immune response can be elicited against different antigens through a single injection. All these findings would also have significance when applied to other infectious and tumor antigens.

## 5. Conclusions

In conclusion, DNA vectors expressing products of fusion between Nef^mut^ and SARS-CoV-2 antigens can be considered candidates for new vaccine strategies aimed at inducing anti-SARS-CoV-2 CTLs. 

## Data Availability

The data presented in this study are available on request from the corresponding author. The data are not publicly available due to patent application.

## Supplementary Materials

The following are available online at https://www.mdpi.com/2076-393X/9/3/240/s1, Figure S1: Raw Western blot data.
