# Supplementary figures and images for: Simultaneous CD8+ T-Cell Immune Response against SARS-Cov-2 S, M, and N Induced by Endogenously Engineered Extracellular Vesicles in Both Spleen and Lungs
Source: Vaccines (Basel). 2021 Mar 10;9(3):240. doi: 10.3390/vaccines9030240 (PMC7999804; doi:10.3390/vaccines9030240)

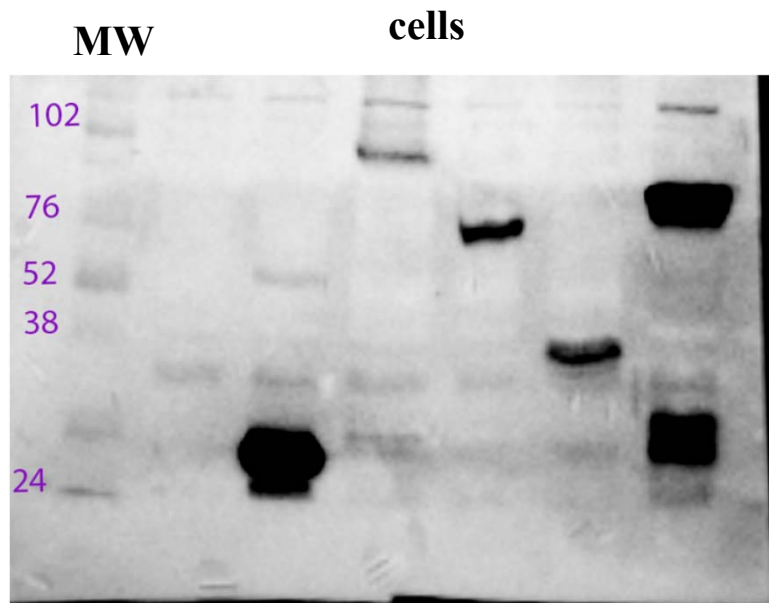

**Anti-Nef**

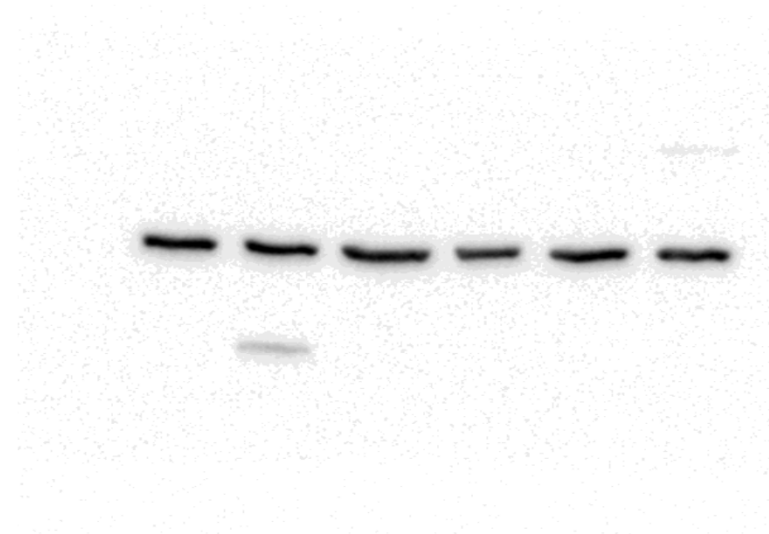

**Anti- $\beta$ -actin**

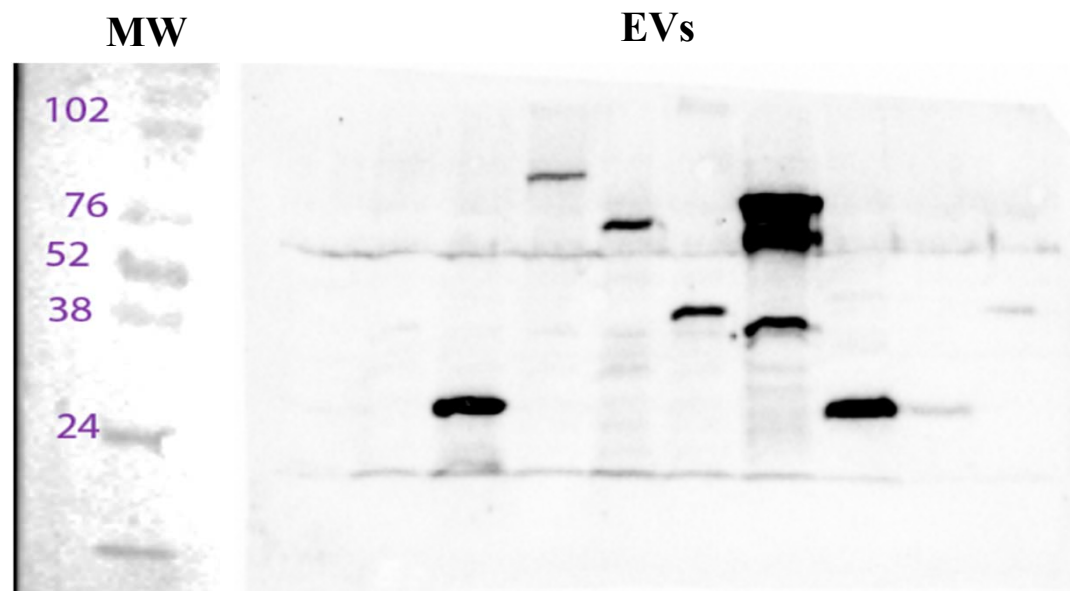

**Anti-Nef**

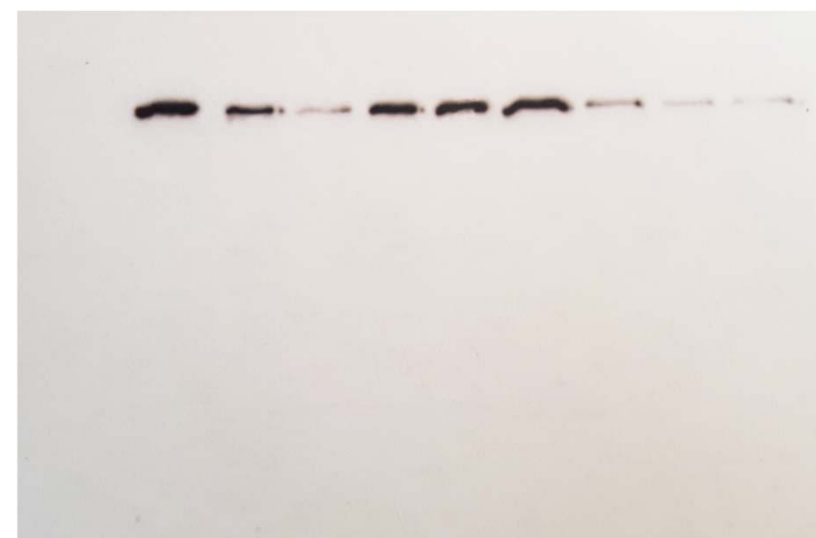

**Anti-Alix**

Supplement: Supplementary file 1 [file vaccines-09-00240-s001.pdf]
